# A single 2000-meter exercise test to assess exercise adaptation in elite rowers during the preparatory phase

**DOI:** 10.3389/fphys.2025.1544637

**Published:** 2025-08-12

**Authors:** Sabina Kaczmarczyk, Anna Kasperska, Hanna Dziewiecka, Joanna Ostapiuk-Karolczuk, Justyna Cichoń-Woźniak, Piotr Basta, Anna Skarpańska-Stejnborn

**Affiliations:** ^1^ Department of Biological Sciences, Faculty of Sport Sciences in Gorzów Wielkopolski, University of Physical Education, Poznań, Poland; ^2^ Department of Physical Education and Sport, Faculty of Sport Sciences in Gorzów Wielkopolski, University of Physical Education, Poznań, Poland

**Keywords:** physical effort, athletes, inflammation, oxidative stress, high-intensity, leukocytosis

## Abstract

**Introduction:**

A 2000-m exercise test on a rowing ergometer is intended to determine the training program’s effectiveness and the proper training intensity. However, anecdotal data from rowers confirm that this test involves extremely high levels of exercise, which may induce metabolic stress. Therefore, this observational study aims to analyze the effects of a 2000-m exercise test on biomarkers of inflammation, oxidative stress, and the immune system during the preparation phase in elite rowers.

**Methods:**

The Polish Youth National Rowing Team participated in the study (N = 18). The rowers performed a 2000-m exercise test on a rowing ergometer. Before the exercise test, after one and 24 h afterwards, venous blood was taken to determine biomarkers. Pro-oxidant-antioxidant balance parameters (total antioxidant capacity, thiobarbituric acid reactive substances), pro- and anti-inflammatory cytokine levels (IL-2, IL-10, TNF-α, IL-6), and MYO were determined. This is part of a study that has been registered on clinicaltrials.gov under the identifier NCT06133751.

**Results:**

A statistically significant increase in IL-6 concentrations was observed 24 h after exercise. IL-10 concentrations statistically decreased significantly immediately after and 24 h post exercise. MYO levels statistically decreased significantly immediately after and 24 h post exercise. WBC statistically increased significantly immediately after exercise and then returned to baseline values.

**Conclusion:**

The results of this study confirm that the 2000-m exercise test causes short-term inflammation, which is related to the physiological recovery process. In addition, chronic inflammation and immune system disturbances were not observed, which means that the athletes showed adaptation to very intense exercise.

## 1 Introduction

Exercise testing is a key tool for determining training recommendations for endurance athletes, as well as for monitoring progress and evaluating performance. The outcome of an exercise test is important for athletes, coaches, and trainers, especially when it translates into sports performance at national and international championships. Furthermore, the data collected during exercise testing offers valuable insights into physiological characteristics and adaptations to controlled exercise loads. Which can be used for the initial selection of athletes (Penichet-Tomas et al., 2023). Recent research has demonstrated that training load monitoring through physiological markers can enhance performance assessment in competitive athletes (Padulo, Manenti, and Esposito, 2023) A non-invasive, practical, and reliable 2000-m test on a rowing ergometer that can accurately predict performance is common in professional rowing (Otter et al., 2015; Lawton, Cronin, and McGuigan, 2013). The 2,000 m is the distance that applies to athletes at national and international competitions. Need for high endurance and aerobic capacity as well as overall muscular strength is related to the maximum oxygen consumption when covering a distance of 2000 m in 5.5–7.0 min (Penichet-Tomas et al., 2023). It is worth noting that rowers use a unique physiological race pace pattern, meaning that they start the effort with a vigorous sprint that places excessive demands on anaerobic metabolism, followed by a very high aerobic state and then a grueling sprint to the finish line. Tolerance to excessive anaerobiosis is evident through very high lactate levels and O2 deficit measured during the first 2 min of exercise (Hagerman et al., 2006). Moreover, the rowing exercise protocol is more demanding than running or cycling because all the external and trunk muscles are engaged during rowing, and rowers have relatively high body mass values (Jürimäe, Purge, and Tillmann, 2021). Furthermore, the continuous, alternating limb movements of cyclists and runners *versus* the simultaneous limb movement with interruption by the rowers during the coiling recovery phase can explain the mechanical efficiency differences (Hagerman et al., 2006). Otter et al. reported that anecdotal evidence from rowers confirms that this test has an extremely high level of exertion. Therefore, this test is not suitable to be performed on a regular basis (Otter et al., 2015).

Given that today’s elite rowing training programs can include up to 14 training sessions per week (Bourdon, David, and Buckley, 2009), the additional performance of a 2000-m exercise test to determine the effectiveness of the training program and the appropriate training intensity may influence the induction of metabolic stress. Monitoring internal and external training load is a challenge for researchers, coaches, and athletes. It is desirable to determine whether exercise-induced perturbations are physiological and related to exercise adaptation.

Exercise adaptation is observed when the right balance between effort and recovery is achieved. High training loads with insufficient recovery periods weaken training adaptation, which can in time, manifest as overtraining (Holt et al., 2019). Moreover, physiological responses to exertion differ between athletes and are influenced not only by factors such as gender or training level but also by different modalities of the same sport (Penichet-Tomas et al., 2023). To our knowledge, there are no studies that have analyzed in detail whether such an intense 2000-m exercise test induces metabolic stress and immune dysfunction and whether it is followed by mechanisms that induce exercise adaptation in elite rowers. Especially if it is performed during the preparatory phase (PREP, i.e., the off-season).

Therefore, this observational study aims to analyze the effects of a 2000-m exercise test on biomarkers of inflammation, oxidative stress, and the immune system. The authors will pay particular attention to the interactions between biomarkers and try to answer whether adaptation to exercise in the preparatory phase is observed in highly trained rowers after an intense test on the rowing ergometer.

## 2 Materials and methods

### 2.1 Participants

**FIGURE 1:**
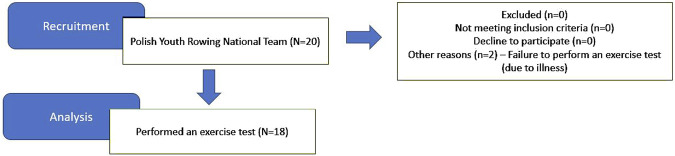
Participant flow.

Twenty men from the Polish Youth National Rowing Team were recruited for the study. However, only eighteen athletes took the 2000-m exercise test (Figure 1). The study was conducted in April during the preparation phase of the training cycle. Before the exercise test, body composition analysis was performed using TANITA MC-780 with an accuracy of 0.05 kg (Tokyo, Japan) (see Table 1). Inclusion criteria for the study included: a) a minimum of 5 years of professional training; b) implementation of the training plan (minimum training time per week 240 min); c) appointment to the Polish youth national rowing team; d) performance of an exercise test (2000-m test on a rowing ergometer). Exclusion criteria included: a) health problems (acute or chronic inflammation, fever, infection); b) use of anti-inflammatory drugs. The experiment was conducted by the Declaration of Helsinki. It is part of a study that was approved by the Ethical Committee of the University of Medical Sciences in Poznan (Resolution No. 390/22) and was registered on clinicaltrials.gov under ID NCT06133751. All participants were informed of the exact conduct of the study and gave written informed consent to participate.

**TABLE 1:** Characteristics of study participants (N = 18).

Parameters	Mean ± SD
Age (years)	20 ± 1.7
Body height (cm)	190.10 ± 4.47
Body mass (kg)	86.35 ± 7.58
Training experience (years)	6.44 ± 1.27

### 2.2 Rowing test

The exercise test was conducted at the beginning of the preparatory phase of an annual training cycle characterized by high volume and training loads. After a 5-min warm-up, the athletes performed a 2000-m exercise test on a rowing ergometer (Concept II, United States). The parameters obtained during the exercise test are shown in Table 2. The athletes performed the test with great commitment and motivation, as their results were decisive for their participation in the championships.

**TABLE 2:** Characteristics of the exercise test.

Parameters	Mean ± SD
Power [watts]	426.77 ± 31.78
W/kg	4.99 ± 0.32
LA_min_ [mmol/L]	2.09 ± 0.56
LA_max_ [mmol/L]	10.48 ± 2.01
Time [seconds]	374.97 ± 8.49

LA, (Lactic Acid); W, (Watts).

### 2.3 Training program

Each day the characteristics of the training units were recorded. Intensity and volume expressed in minutes and type of exercise: a) specific: i.e., rowing (endurance, technique, speed, etc.), b) non-specific: running, strength. Training intensity was also defined by lactic acid (LA) threshold (4 mmol/L): extensive (below LA threshold) or intense (above LA threshold) workload (Table 3).

**TABLE 3:** Training schedule week before exercise test.

Days before the exercise test	I	II	III	IV	V	VI	VII
Time rower, min/day	106	-	150	110	80	100	40
Distance rowed, km/day	28	-	30	20	18	20	8
Training for force development, min/day	-	-	70	-	80	-	-
Extensive endurance rowing training time, min/day	80	-	130	65	80	100	40
High-intensity endurance rowing training time, min/day	26	-	20	45	-	-	-
Unspecific training (running, etc.), min/day	30	80	20	10	30	10	10
Total training time, min/day	136	80	240	120	190	110	50

### 2.4 Material collection and examination

**FIGURE 2:**
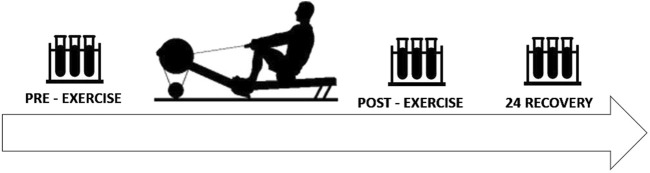
Schematic illustration of the study design.

Three collections were performed: PRE (before the exercise test), POST (immediately after the exercise test), and REST (24 h after the exercise test) (see Figure 2). Blood samples were collected from the ulnar vein into 9 mL tubes (to obtain serum) and into 2.7 mL tubes (to determine morphology). Samples with serum were centrifuged at 2,500 rpm for 10 min. Morphology was determined the same day samples were collected, while serum was frozen and stored at −80°C until testing. Before and immediately after the exercise test, capillary blood was also collected from the earlobe to determine lactate levels.

White blood cells (WBC), such as lymphocytes (LYM), monocytes (MON), granulocytes (GRA) were analyzed using the MYTHIC 18 hematology analyzer (Orphee Medical, Geneva, Switzerland).

Interleukins: IL-2, IL-6, IL-10, TNF-α, and pro-oxidant-antioxidant balance parameters: TAOC, TBARS, and MYO levels were determined using ELISA kits (SunRed Biotechnology Company). TAOC with an assay range of 0.7 U/ml-85 U/ml, TBARS with an assay range of 0.5 ng/mL-100 ng/mL, IL-2 with an assay range of 0.8 ng/L-200 ng/L, IL-6 with an assay range of 1 ng/L-300 ng/L, IL-10 with an assay range of 10 pg/mL-3000 pg/mL, TNF- α with an assay range of 3 ng/L-900 ng/L, MYO with an assay range of 1 ng/mL-300 ng/mL. LA levels were determined immediately after capillary blood sampling using Vario Photometer II (Diaglobal, Berlin, Germany). The concentration of lactate was determined in mmol/L.

### 2.5 Statistical analysis

Statistical analysis and data visualization were performed using GraphPad Prism 9 (GraphPad Software, Boston, United States). Descriptive statistics (mean ± standard deviation, SD) were used to summarize the data and visualize trends across the three time points: pre-exercise, post-exercise, and 24-h rest. Prior to analysis, the Shapiro–Wilk test was applied to assess the normality of distribution, and the Brown–Forsythe test was used to evaluate homogeneity of variances. In addition, outlier detection was conducted using the ROUT method (Q = 1%), and no statistical outliers were identified. For data that met the assumptions of normality and equal variance, differences between the three time points were assessed using a one-way repeated-measures ANOVA, followed by Tukey *post hoc* test for multiple comparisons. For non-normally distributed variables, the non-parametric Friedman test was applied, with Dunn’s multiple comparisons test used as a *post hoc* procedure. Effect sizes were calculated using Cohen’s d for paired comparisons. Based on Cohen’s conventional thresholds, effect sizes were interpreted as small (0.2), medium (0.5), and large (0.8) (Cohen, 1988). In line with reporting standards, we have indicated effect sizes alongside p-values to provide better context for the magnitude of observed changes.

### 2.6 Sample size

Representative study population - sample size was calculated using G-power software. Specifically, we used an effect size derived from the study by Cichoń-Woźniak et al. (2025): https://pmc.ncbi.nlm.nih.gov/articles/PMC12223051/in which IL-6 changes were analyzed in the same training phase of sports preparation. The reported effect size for IL-6 in was Cohen’s d = 1.06 (mean from three calculations, Cohen’s f was calculated from the equation f = d/2 = 0.53 (Ellis, P. D. 2010). The essential guide to effect sizes: Statistical power, meta-analysis, and the interpretation of research results. Cambridge university press.).

Accordingly, we conducted an *a priori* power analysis in G*Power for repeated-measures ANOVA (within factors), using the following parameters:

• Effect size (f) = 0.53.
• Power (1–β) = 0.8.
• α = 0.05.
• Correlation among repeated measures = 0.5.
• Nonsphericity correction ε = 0.5.

This yielded a required minimum sample size of 12 participants, which we exceeded by recruiting 18. Furthermore, the strong IL-6 response observed in our data is consistent with a previous meta-analysis, which also reported large effect sizes for IL-6 increases in response to intense exercise.

## 3 Results

### 3.1 WBC indices

WBC counts increased significantly immediately after exercise (p < 0.0001, Cohen’s d = 2.66; PRE-exercise vs POST-exercise) and then decreased significantly 24 h after the exercise test (p < 0.0001, Cohen’s d = 3.11; POST-exercise vs 24 h REST). Monocytes and granulocytes follow the same trend. An increase was observed immediately after exercise in MON (p = 0.0019, Cohen’s d = 1.08; PRE-exercise vs POST-exercise) and in GRA (p < 0.0001, Cohen’s d = 1.12; PRE-exercise vs POST-exercise) and then a significant decrease 24 h after the exercise in MON (p < 0.0001, Cohen’s d = 2.95; POST-exercise vs 24 h REST) and in GRA (p = 00,046, Cohen’s d = 1.00; POST-exercise vs 24 h REST). The count of LYM significantly increased immediately after exercise (p < 0.0001, Cohen’s d = 1.62; PRE-exercise vs POST-exercise) and then decreased significantly 24 h after the exercise test (p < 0.0001, Cohen’s d = 3.89; POST-exercise vs 24 h REST and p < 0.0001, Cohen’s d = 1.08; PRE-exercise vs 24 h REST) (Table 4).

**TABLE 4:** White blood cell values.

	PRE-exercise	PRE/POST	POST-exercise	POST/REST	24 h REST	PRE/REST
WBC [10^3^/μL]	5.53 ± 1.01	p < 0,0001 Cohen’s d = 2.66	8.42 ± 1.16	p < 0,0001 Cohen’s d = 3.11	5.12 ± 0.963	p = 0,1053 Cohen’s d = 0.42
LYM [10^3^/μL]	2.50 ± 0.52	p < 0,0001 Cohen’s d = 1.62	4.07 ± 0.62	p < 0,0001 Cohen’s d = 3.89	1.97 ± 0.46	p < 0,0001 Cohen’s d = 1.08
MON [10^3^/μL]	0.40 ± 0.07	P = 0,0019 Cohen’s d = 1.08	0.61 ± 0.10	p < 0,0001 Cohen’s d = 2.95	0.33 ± 0.09	p = 0,4697 Cohen’s d = 0.40
GRA [10^3^/μL]	2.62 ± 0.86	p < 0,0001 Cohen’s d = 1.12	3.73 ± 1.13	p < 0,0001 Cohen’s d = 1.00	2.73 ± 0.88	p = 0.73 Cohen’s d = 0.13

WBC, (white blood cells); LYM, (lymphocytes); MON, (monocytes); GRA, (granulocytes). Values are presented as mean ± SD.

### 3.2 Pro- and anti-inflammatory markers, and muscle damage

**FIGURE 3:**
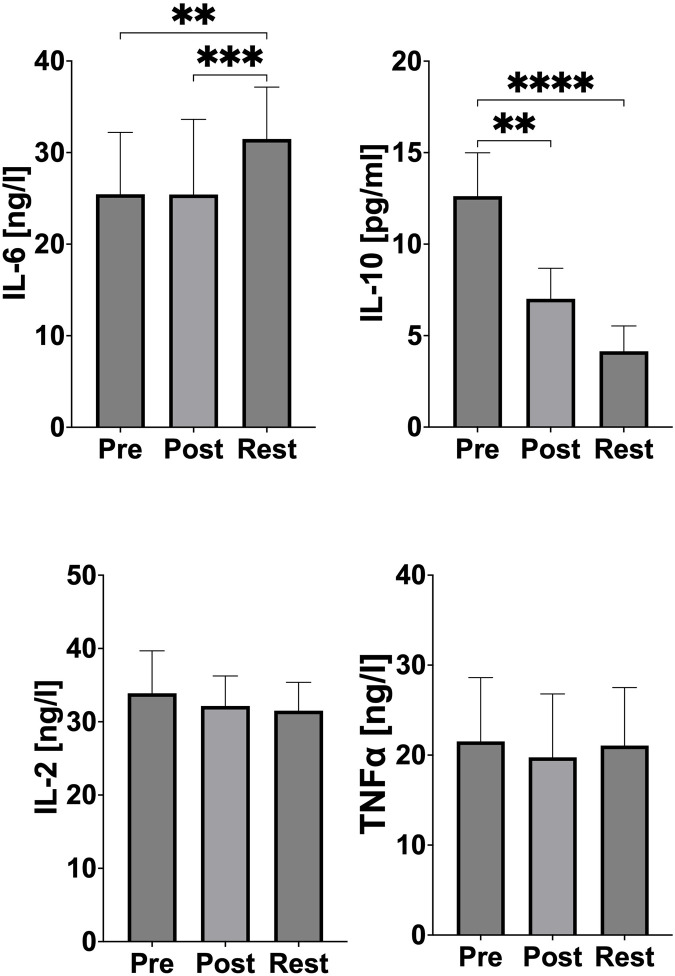
Changes of pro- and anti-inflammatory markers during acute exercise. PRE (pre-exercise), POST (post-exercise), REST (24 h recovery), IL-6 (interleukin 6), IL-10 (interleukin 10), IL-2 (interleukin 2), TNF-α (tumor necrosis factor). Significant differences: *p < 0.05, **p < 0.01, ***p < 0.001, ****p < 0.0001.

A statistically significant increase in IL-6 concentration was observed 24 h after exercise (p = 0.0046, Cohen’s d = 0.97; PRE-exercise vs 24 h REST and p = 0.0004, Cohen’s d = 0.88; POST-exercise vs 24 h REST). In the case of IL-10, a statistically significant reduction was observed immediately after exercise (p = 0.0014, Cohen’s d = 2.79; PRE-exercise vs POST-exercise) and followed by an even greater reduction 24 h after the exercise test (p < 0.0001, Cohen’s d = 3.66; PRE-exercise vs 24 h REST). No significant changes in IL-2 and TNF-α concentration were observed (Figure 3).

**FIGURE 4:**
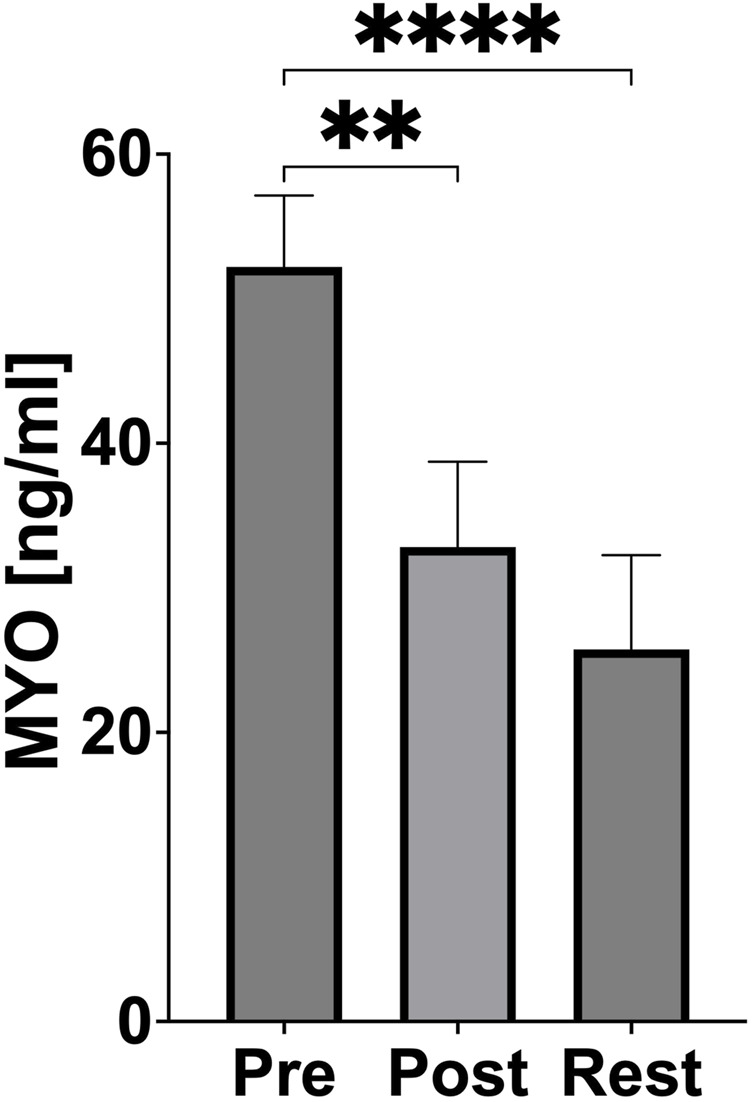
Changes of muscle damage during acute exercise. PRE (pre-exercise), POST (post-exercise), REST (24 h recovery), MYO (myoglobin). Significant differences: *p < 0.05, **p < 0.01, ***p < 0.001, ****p < 0.0001.

Myoglobin concentration significantly decreased immediately after exercise (p = 0.0014, Cohen’s d = 3.58; PRE-exercise vs POST-exercise) and an even greater reduction was subsequently observed 24 h after exercise (p < 0.0001, Cohen’s d = 4.62; PRE-exercise vs 24 h REST) (Figure 4).

### 3.3 Markers of oxidative stress and antioxidant defense

**FIGURE 5:**
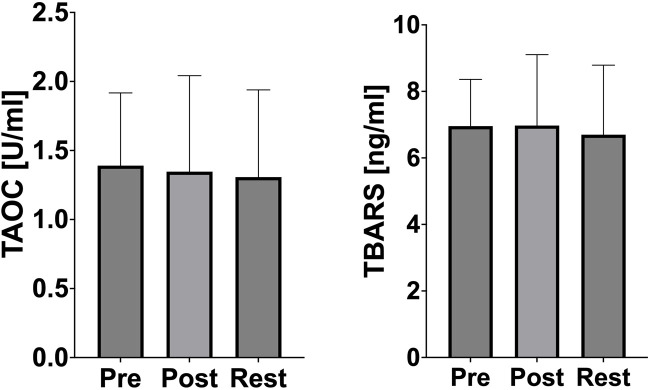
Changes of oxidative stress marker and antioxidant defense during acute exercise. PRE (pre-exercise), POST (post-exercise), REST (24 h recovery), TAOC (total antioxidant capacity), TBARS (thiobarbituric acid reactive).

No significant changes in TAOC and TBARS concentration were observed (Figure 5).

## 4 Discussion

To our knowledge, no studies have assessed the effects of an intense 2000-m rowing ergometer test on biomarkers of inflammation, oxidative stress, and immune response in elite rowers. However, several studies have analyzed specific individual indicators, and the 2000-m test is often employed as a stressor to evaluate the effectiveness of supplementation or physiological adaptations in elite athletes. The results of this study, interpreted in the context of other research, provide evidence of effective physiological adaptation of rowers to high-intensity exercise and offer insights into their advanced training status.

In the current study, IL-6 concentrations increased significantly 24 h after the exercise test compared to both the resting and immediate post-exercise states (Figure 3). This suggests the activation of inflammatory pathways, muscle microdamage repair mechanisms, and metabolic stress responses. The increase in IL-6 is a typical inflammatory reaction and a marker of the physiological defense mechanism in response to intense exercise. Importantly, the delayed peak of IL-6 at 24 h, rather than immediately post-exercise, may indicate the controlled release of this cytokine from leukocytes as part of a well-regulated recovery process. Jürimäe et al. similarly reported an increase in IL-6 following long-distance rowing in male elite rowers, linking this response to the acute inflammatory processes that facilitate muscle adaptation and repair. Additionally, the authors indicate that circulating IL-6 during acute exercise is also a metabolic marker of exercise stress and is mainly produced by working skeletal muscle tissue in relation to glycogen levels (Jürimäe et al., 2021). The slight IL-6 response in our study, compared to longer-duration exercises, likely reflects the athletes’ advanced conditioning, which minimizes excessive inflammatory activation. It is also worth noting that IL-6 may originate not only from skeletal muscle fibers, but also from other tissues, such as intramuscular connective tissue, tendons/ligaments, and vascular endothelium. This heterogeneity is a limitation when muscle biopsies are not used to directly assess IL-6 mRNA expression in skeletal muscle after exercise (Langberg et al., 2002). Furthermore, it should be noted that tissues rich in fibroblasts can significantly contribute to the release of IL-6 and potentially exhibit pronounced adaptive responses (Skutek et al., 2001). Unfortunately, due to methodological constraints, we were unable to assess IL-6 expression at the tissue-specific level or evaluate fibroblast-specific parameters directly in this study.

IL-6 also plays a significant role in mobilizing immune responses. In our study, a statistically significant increase in white blood cell indices (including leukocytes, monocytes, and granulocytes) was observed immediately after the exercise test, returning to baseline within the recovery period (Table 4). This transient leukocytosis is a hallmark of physiological immune mobilization, supporting the body’s preparedness to counteract exercise-induced microtrauma and potential infection. This phenomenon, often referred to as “exercise-induced leukocytosis,” is a sign of a healthy immune system capable of acute activation without progression to pathological inflammation. (Bizjak et al., 2021; Minuzzi et al., 2019; Kuliczkowski et al., 1997). The rapid normalization of leukocyte subtypes observed in this study suggests efficient immune regulation and recovery in the tested athletes, consistent with their training adaptations. Although a decrease in lymphocytes relative to baseline was observed in this study, there is no ‘lymphocytopenia’. This phenomenon assumes clinically low reference level values (<1.0 × 109/L), falling 30%–50% below pre-exercise values and able to remain reduced up to 6 h later (Simpson et al., 2015). Although lymphocyte downregulation during recovery may temporarily increase susceptibility to infection, the rapid recovery of other immune markers highlights the effective balance between inflammatory responses and athlete recovery (Minuzzi et al., 2019; Krüger and Mooren, 2007).

An important finding was the reduction in IL-10 concentrations, both immediately post-exercise and at 24 h (Figure 3). IL-10 is an anti-inflammatory cytokine that modulates inflammatory responses. The reduced IL-10 levels in our study may indicate a well-controlled inflammatory process that does not require excessive anti-inflammatory suppression. This finding is consistent with the hypothesis that a balanced inflammatory response allows for effective tissue repair and immune activation without impairing the natural physiological response to exercise (Juszkiewicz et al., 2019). While studies by Jürimäe et al. showed an increase in IL-10 after prolonged exercise in rowers, these results were associated with longer exercise durations (2 h), which may elicit a greater need for anti-inflammatory regulation (Jürimäe et al., 2021; Jürimäe et al., 2016). This is also confirmed by Cabral-Santos et al., who conducted a systematic review in which they analysed twelve scientific studies related to changes in IL-10 levels after physical exercise. The researchers conclude that the duration of exercise is the most important factor determining the magnitude of the exercise-induced increase in plasma IL-10 concentrations (Cabral-Santos et al., 2019). The individual variability of the rowers studied should also be taken into account. This therefore provides scope for analysis in future studies to confirm or rule out similar results.

In contrast, the moderate reduction in IL-10 observed may reflect the rowers’ adaptation to shorter, high-intensity efforts, reducing the need for prolonged anti-inflammatory mechanisms while promoting cellular immunity.

The absence of changes in TNF-α and IL-2 concentrations (Figure 3) further supports the hypothesis that the post-exercise inflammatory response in this study did not progress into a chronic inflammatory phase. Chronic inflammation is often a marker of overtraining or insufficient recovery. The stability of these cytokines demonstrates the athletes’ capacity to handle the physiological demands of the test without adverse systemic effects, highlighting their good adaptation to high-intensity exercise. Similar results were reported by Jürimäe et al. in female elite rowers after prolonged exercise (Jürimäe et al., 2018), as well as in our previous research on male elite rowers (Skarpańska-Stejnborn et al., 2014). These findings suggest that regular exposure to high training loads in elite athletes induces a state of physiological resilience, minimizing the risk of chronic inflammation (Skarpańska-Stejnborn et al., 2014; Jürimäe et al., 2018).

Muscle damage, as indicated by myoglobin levels, was not pronounced in this study. Furthermore, bilateral neuromuscular responses to high-intensity exercise have been shown to vary with exercise velocity, which may influence the interpretation of muscle damage markers in rowing athletes (Padulo et al., 2022). A significant reduction in myoglobin concentration was observed immediately after exercise, with further decreases at 24 h, suggesting efficient muscle regeneration and adaptation (Figure 4). Hansen et al. reported increases in myoglobin after prolonged, intense exercise in both rowers and runners, particularly in less-conditioned athletes (Hansen et al., 1982). The observed reduced myoglobin response in our study highlights the effectiveness of athlete training in minimising exercise-induced muscle damage. This adaptation likely reflects enhanced muscle repair mechanisms and a well-developed tolerance to eccentric muscle contractions, reducing the risk of injury during repeated high-intensity efforts. The observed decrease in myoglobin concentration following physical exercise is unexpected and may be attributable to measurement variability. Repetition of myoglobin measurements in future studies is recommended to assess the reproducibility and validity of this finding.

Oxidative stress markers, such as thiobarbituric acid reactive substances (TBARS) and total antioxidant capacity (TAOC), remained stable throughout the study, further emphasizing the athletes’ advanced training status (Figure 5). The lack of oxidative stress suggests that the rowers possess a robust antioxidant defense system capable of neutralizing exercise-induced free radicals. Previous studies confirm that long-term endurance training increases the activity of both enzymatic (e.g., superoxide dismutase, glutathione peroxidase) and non-enzymatic (e.g., vitamins C and E, glutathione) components of the antioxidant system, potentially providing sufficient protection against oxidative stress, which is usually caused by intense physical exertion (Miyazaki et al., 2001). Dernbach et al. reported similar findings, demonstrating the absence of oxidative stress in well-trained rowers after prolonged exercise (Dernbach et al., 1993). Additionally, the absence of oxidative stress in the present study aligns with findings by Cubrilo et al., who noted that trained athletes exhibit lower susceptibility to oxidative damage due to efficient mitochondrial function and antioxidant enzyme activity (Cubrilo et al., 2011). These results highlight the athletes’ metabolic efficiency and resilience to oxidative stress, even under high-intensity exercise conditions.

## 5 Conclusion

**FIGURE 6:**
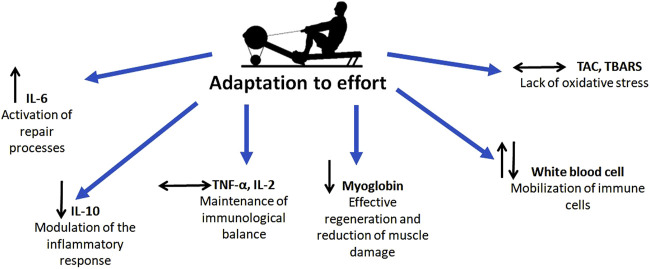
Schematically presented research conclusions.

In summary, the results of this study demonstrate that the 2000-m rowing ergometer test induces a physiological inflammatory response (as indicated by increased IL-6) and immune activation (as indicated by leukocytosis), while maintaining efficient recovery mechanisms. The absence of chronic inflammation (evidenced by stable TNF-α and IL-2 levels), the reduction in muscle damage markers, and the stability of oxidative stress indicators collectively highlight the rowers’ excellent adaptation to high-intensity exercise (Figure 6). These findings confirm that the tested athletes possess a well-developed capacity to manage and recover from intense physical efforts, reflecting the effectiveness of their training regimens. This study underscores the importance of regular, structured training in enhancing physiological resilience and reducing the risk of maladaptive responses to high-intensity exercise.

## 6 Limitations

The most important limitation of this study is the relatively small study group. Conducting research in elite sports presents challenges in gathering a larger group of highly trained athletes with comparable performance levels and training patterns. Another limitation was the unexpectedly large effect size. A further limitation arises from the high inter-individual variability in biomarker concentrations, with measurements of these markers varying according to gender, age and training level. Subsequently, exercise-induced responses vary between sports, and by duration and type of exercise. Another limitation stems from the measurement methodology. Sampling after 24 h may not have captured the peak of inflammatory biomarkers, which occur approximately 6–12 h after exercise.

## Data Availability

The original contributions presented in the study are included in the article/supplementary material, further inquiries can be directed to the corresponding author.
